# Lipid Metabolism in Neurons: A Brief Story of a Novel c-Fos-Dependent Mechanism for the Regulation of Their Synthesis

**DOI:** 10.3389/fncel.2019.00198

**Published:** 2019-05-07

**Authors:** Lucia Rodríguez-Berdini, Beatriz L. Caputto

**Affiliations:** Centro de Investigaciones en Química Biológica de Córdoba (Consejo Nacional de Investigaciones Científicas y Técnicas), Departamento de Química Biológica “Ranwel Caputto”, Facultad de Ciencias Químicas, Universidad Nacional de Córdoba, Córdoba, Argentina

**Keywords:** nervous system phospholipid synthesis regulation, membrane biogenesis, proliferation and differentiation, brain tumors, endomembrane compartments

## Abstract

The mechanisms that coordinately regulate lipid synthesis in the nervous system together with the high rates of membrane biogenesis needed to support cell growth are largely unknown as are their subcellular site of synthesis. c-Fos, a well-known AP-1 transcription factor, has emerged as a unique protein with the capacity to associate to specific enzymes of the pathway of synthesis of phospholipids at the endoplasmic reticulum and activate their synthesis to accompany genomic decisions of growth. Herein, we discuss this effect of c-Fos in the context of neuronal differentiation and also with respect to pathologies of the nervous system such as the development and growth of tumors. We also provide insights into the sub-cellular sites where this regulation occurs at the endoplasmic reticulum membranes and the molecular mechanism by which c-Fos exerts this activity.

## Introduction

Lipids, essential constituent molecules of all organisms, participate in a broad range of cellular processes. Their importance is highlighted by the fact that almost 5% of the human genes are related to their synthesis (van Meer et al., 2008). Particularly, the nervous system has a rich lipid composition, being the brain the second tissue in humans with both the highest lipid content and the highest diversity in their composition (Sastry, 1985; Hamilton et al., 2007; Bozek et al., 2015). Some authors have related this diversity to the cognitive abilities acquired by humans through their evolutionary lineage (Bozek et al., 2015). Among the main functions of lipids in the nervous system, of undoubtable importance is their structural role in biological membranes, their participation as bioactive messengers involved in cell signaling and their contribution to energy supply (Tracey et al., 2018).

The homeostasis of the lipid content of the nervous system is of vital importance, since several neuropathologies have been associated to their abnormal metabolism (Ahmed et al., 2017; Gaspar et al., 2018; Valadas et al., 2018; Yu et al., 2018). Evidence has linked disturbances in cholesterol metabolism to the progressive neurodegeneration observed in Alzheimer patients (Chang et al., 2017; Habchi et al., 2018; Penke et al., 2018) and also to autism spectrum disorders, possibly through lipid rafts disarrangements and the consequent alteration of synaptic functions (Wang, 2014). Elevated ganglioside levels have been related to Parkinson’s disease, probably through their influence on α-synuclein aggregation kinetics (Gaspar et al., 2018). Abnormalities in the metabolism of polyphosphoinositides also underlie nervous system diseases such as schizophrenia, bipolar disorder, Friedrich’s ataxia, Parkinson’s and Down syndrome (Lauwers et al., 2016).

An unbalanced content of fatty acids in the nervous system has also been associated to neurodegenerative diseases. Docosahexaenoic acid (DHA) and arachidonic acid (AA) are the most abundant and biologically active essential fatty acids in brain phospholipids (Sanchez-Mejia and Mucke, 2010). DHA and its derivatives, the docosanoids, are involved in neuronal survival signaling pathways that resolve inflammation and reduce oxidative stress (Farooqui, 2012; Bazan, 2014; Tayebati, 2018). A significant decrease in DHA levels has been observed in cognitive impairment and Alzheimer’s disease (AD) (Bazan et al., 2011; Bazan, 2014; Kim et al., 2014). In fact, DHA enriched diets have been proven to be efficient against neurodegeneration and able to prevent AD symptoms (Strokin et al., 2006; Hooijmans et al., 2009; Oster and Pillot, 2010; Tayebati, 2018). In retina neurons, DHA protects cells against oxidative stress and promotes photoreceptor differentiation (Simon et al., 2016). On the other hand, an activated metabolism of AA and its derivatives, the eicosanoids, has been associated to different pathologies, such as multiple sclerosis or AD, probably through the promotion of neuroinflammation and of an increase in neuronal activity and excitotoxicity (Lukiw and Bazan, 2000; Sanchez-Mejia and Mucke, 2010; Palumbo, 2017).

Brain choline metabolism has received much attention due to the important role of acetylcholine transmission in neuronal functions, especially those involved in cognitive abilities (Ferreira-Vieira et al., 2016). Abnormalities in the brain cholinergic system have been observed in AD, Lewy Bodies Dementia, Parkinson’s disease and Huntington, among other pathologies (Pepeu and Grazia Giovannini, 2017).

In the case of the neuronal membrane, its lipid composition varies according to the neuronal type and the structural compartment of the neuron (Vance et al., 2000). The first results obtained after addressing the subcellular localization of lipid synthesis in neurons indicated that it was carried out in the neuronal soma (Weiss and Hiscoe, 1948), from where these components were transported by anterograde transport to the growing dendrites and axons during differentiation (Ledeen, 1985). However, it was later suggested that axonal transport was neither fast nor abundant enough to supply the necessary amounts of lipids to support axonal membrane expansion, an event that can reach an increment of up to 20% per day during the peak of neuronal growth (Pfenninger, 2009). So, given the size and architecture of neurons, it seemed reasonable to imagine the existence of a temporal and domain-specific organization. This could regulate lipid synthesis at distal sites of the neuron so as to avoid the sole dependence on axonal transport of these compounds from the soma.

Several reports have demonstrated the presence in axons of endoplasmic reticulum (ER) membranes, the main organelle for lipid synthesis, suggesting that the axon could contain a local functional biosynthetic machinery (Palay, 1958; Tsukita and Ishikawa, 1976; Rambourg and Droz, 1980; Lindsey and Ellisman, 1985; Gonzalez and Couve, 2014; Luarte et al., 2018; Terasaki, 2018). In fact, this seems to be the case: several enzymes of the pathway of lipid synthesis have been shown to be present in axons where they are capable of locally synthetizing phosphatidylcholine, phosphatidylethanolamine, phosphatidylserine, phosphatidylinositol, sphingomyelin and fatty acids (Vance et al., 1991, 1994). For example, 50% of the phosphatidylcholine required for normal axonal elongation has been shown to be locally synthetized in the axonal ER (Posse de Chaves et al., 1995).

These observations posed questions regarding the mechanisms that determine which lipids are synthetized in the axon, the quantitative extent of the domain-specific synthesis of lipids and the regulatory constraints driving this phenomenon. In this regard, many aspects of this metabolism remain unanswered, especially if its influence on neuronal plasticity is considered.

## c-Fos: One Protein Leading to at Least Two Roads

The oncogene *fos* codifies for a protein of 380 amino acids called c-Fos that belongs to the *Immediate Early Genes* (IEGs) family (Angel and Karin, 1991). Five different proteins form the Fos family: c-Fos, Fos-B, ΔFos-B, Fra-1, and Fra-2 (Tulchinsky, 2000; Raivich and Behrens, 2006), all of which heterodimerize mainly with proteins of the Jun family thus forming the well-known AP-1 family of transcription factors (Angel and Karin, 1991). c-Fos heterodimerizes with Jun through a leucine zipper domain (LZ) and the resulting heterodimer interacts with a DNA consensus sequence through the basic domains (BD) of both proteins (Glover and Harrison, 1995).

It has been established that AP-1 activity is induced under a wide range of physiological and pathological stimuli including cytokines, growth factors, stress signals, infections and upon oncogenic transformation (Shaulian and Karin, 2001). It has also been proposed that its activity can be regulated through multiple pathways: (a)- via the transcriptional regulation of the proteins that compose each AP-1 dimer; (b)- through the stability of their mRNAs; (c)- via post-transductional modifications; (d)- by regulating the turnover of pre-existing molecules of the proteins or of the newly synthetized ones; (e)- through specific interactions between the different AP-1 subunits; (f)- through interactions with other transcription factors and co-factors (Hess et al., 2004).

Under certain circumstances, c-Fos is capable of inducing cellular transformation (Miller et al., 1984), a role that is usually associated to its function as an AP-1 transcription factor. Evidence has been provided that demonstrates its main role in cell growth and in neoplastic transformation (Tulchinsky, 2000; Shaulian and Karin, 2001; Hess et al., 2004; Milde-Langosch, 2005). Surprisingly, it was shown that, unlike other oncogenes studied, its oncogenic activity does not rely on mutations of its sequence but is rather dependent only on its over-expression (Miller et al., 1984). In addition, different stress signals such as UV exposure or alkylating agents, that normally provoke cell cycle arrest or cell death, also induce its expression (Buscher et al., 1988; Curran and Franza, 1988; Piechaczyk and Blanchard, 1994; Shaulian and Karin, 2001). Even more, numerous studies pointed out that the outcome of AP-1 activity seems to be cell-type specific: whereas in some cells it promotes apoptosis, in others it is required for survival (Hess et al., 2004). Hence, although the different functions of AP-1 have been exhaustively studied, the complete scenario resulting from its expression/overexpression is complex due to its capacity to exert completely antagonizing functions. This duality can be particularly distinguished in the nervous system where AP-1 is involved in both degenerative and regenerative processes, as a degeneration effector or as a physiological neuroprotective player (Herdegen and Waetzig, 2001).

In the nervous system, c-Fos expression has also been related to processes involving memory and learning (Dragunow, 1996; Grimm and Tischmeyer, 1997; Rosen et al., 1998; Minatohara et al., 2015; Gallo et al., 2018). These findings transformed this protein into a marker of plastic changes that favor the establishment of long term memory and the maintenance of specific neuronal populations (Rosen et al., 1998; Minatohara et al., 2015). In fact, AP-1 inhibition negatively affects memory formation and physiological adaptive processes such as the synchronization of the endogenous clock (Wollnik et al., 1995; Grimm and Tischmeyer, 1997). While all this evidence has reliably demonstrated the importance of c-Fos and the functional consequences of its induction, surely other cellular mechanisms in which this protein participates, remain unknown.

**FIGURE 1:**
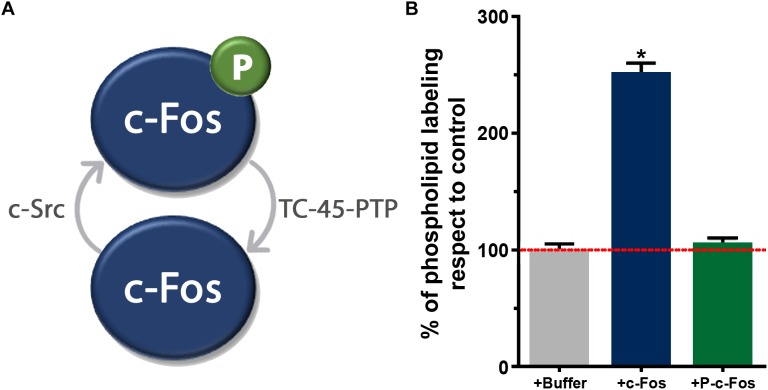
**(A)** Schematic representation of the phosphorylation/dephosphorylation cycle of the tyrosine residues of c-Fos by the kinase c-Src and the phosphatase TC-45-PTP. Dephosphorylated c-Fos is able to associate to ER membranes whereas phosphorylated c-Fos is not. **(B)** Labeling of total phospholipids in T98G cells homogenates in the presence of recombinant dephosphorylated c-Fos and phosphorylated c-Fos (obtained after incubation with recombinant c-Src). The capacity to incorporate [^32^P] to phospholipids from [^32^P]-ATP was measured *in vitro*. The results are the mean ± SD of two experiments performed in triplicate. ^∗^*p* < 0.002 with respect to the control, as determined by Student’s two-tailed *t*-test. Adapted from Ferrero et al., 2012 (ownership of copyright in original Springer Nature research articles remains with the Author, and provided that, when reproducing the contribution or extracts from it or from the Supplementary Information, the author acknowledges first and reference publication in the Journal).

During the last 20 years, our laboratory has accumulated evidence that demonstrates that c-Fos is a moonlighting protein: it is capable of performing two different, apparently autonomous, unrelated functions. In addition to its well-known function as an AP-1 transcription factor, c-Fos associates to the ER where it activates lipid synthetizing enzymes independently of its nuclear function (Guido et al., 1996; Bussolino et al., 1998, 2001; Gil et al., 2004; Crespo et al., 2008; Alfonso Pecchio et al., 2011; Caputto et al., 2014; Cardozo Gizzi et al., 2015). This phenomenon has been observed in animal models with nervous system tumors, in cultured cells derived from the nervous system (PC12 cells, the neuronal tumor cell lines T98G, U87MG, NB41A3, C6, among others) and in non-nervous cells such as breast cancer cells (Gil et al., 2004; Motrich et al., 2013). The association of c-Fos to the ER is essential for lipid synthesis activation to occur and it is regulated by a phosphorylation/dephosphorylation cycle exerted by the enzymes c-Src and TC45-PTP. c-Src phosphorylates the tyrosine residues 10 and 30 of c-Fos determining its dissociation from the ER. By contrast, TC45-PTP is the phosphatase that removes the phosphate groups allowing c-Fos to associate to membranes in its dephosphorylated form (Figure 1; Ferrero et al., 2012).

**FIGURE 2:**
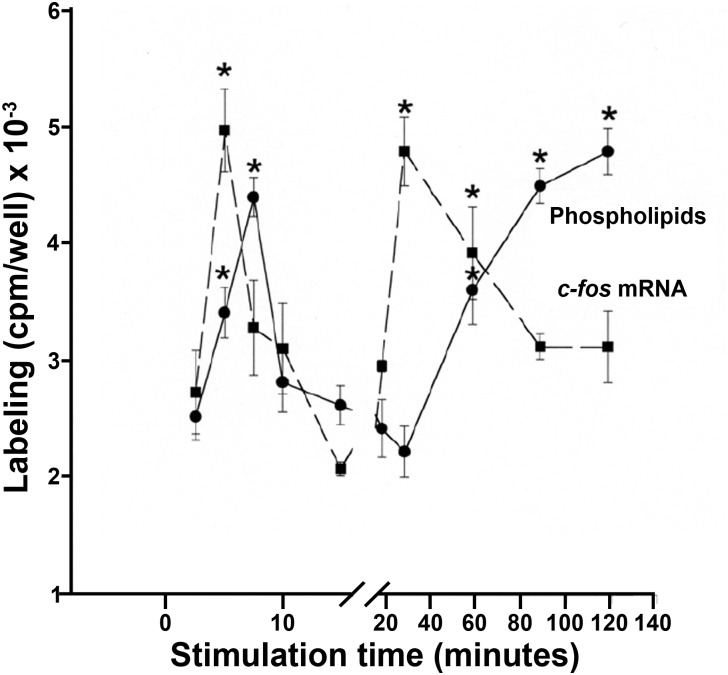
Time curves of phospholipid metabolic labeling (circles, full line) and c-Fos mRNA expression (squares, dashed line) in NIH-3T3 cells stimulated to re-enter cell cycle after quiescence. In all the conditions, phospholipid labeling was determined by pulsing cells with ^32^P-orthophosphate during 15 min, irrespective of the stimulation status. The results are the mean ± SD of six independent experiments performed in duplicate. ^∗^*p* < 0.025 with respect to time 0 of stimulation. Adapted from Bussolino et al., 2001 (the Copyright Clearance Center, Inc. grants authors rights to reproduce figures or tables from their own works free of charge).

**FIGURE 3:**
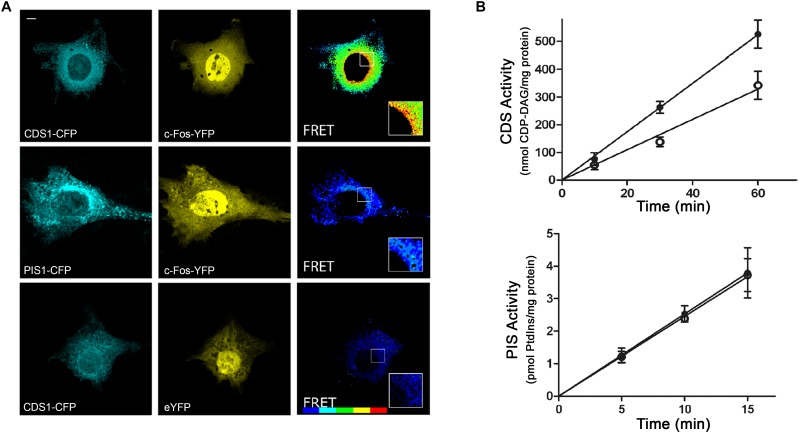
The enzyme CDP-diacylglycerol synthase (CDS) interacts with and is activated by c-Fos, while the enzyme phosphatidylinositol synthase (PIS) neither interacts nor is activated by c-Fos. **(A)** FRET experiments of cells co-transfected to express c-Fos-YFP and CDS1-CFP (first row) or PIS1-CFP (second row) were examined by confocal microscopy using filters for eCFP (left) or for eYFP (middle). FRET efficiency images were obtained and pseudocolored (right). The last row shows control cells co-expressing CDS1-CFP and eYFP. FRET scale goes from no FRET (blue) to maximum FRET (red). Scale bar: 5 μm. **(B)** Effect of c-Fos on CDS and PIS activities. Assays were performed in the presence (black circles) or the absence (open circles) of recombinant c-Fos. Formation of CDP-diacylglycerol (CDP-DAG) was measured in the case of determining CDS activity and phosphatidylinositol (PtdIns) in the case of examining PIS activity. Results are the mean ± SD of at least two experiments performed in triplicate. Adapted from Alfonso Pecchio et al., 2011 (article available to the public under an Attribution–Noncommercial–Share Alike 3.0 Unported Creative Commons License - http://creativecommons.org/licenses/by-nc-sa/3.0).

Using different types of cells in culture, we showed that the expression of c-Fos mRNA exhibits two peaks, each of them concordant with a significant increase in the metabolic labeling of lipids (Figure 2; Bussolino et al., 2001). Specifically blocking c-Fos expression also blocks activation of lipid synthesis. Even more, in order to obtain an insight on the mechanism for enzyme activation, we performed biochemical assays. We found that at the ER, c-Fos is capable of activating only some particular enzymes of the different lipid synthetizing pathways: CDP-diacylglycerolsynthase-1 (CDS1), Phosphatidylinositol-4-kinase type II α (PI4KIIα) and Lipin-1 are activated by c-Fos, whereas Phosphatidylinositol synthase (PIS1) and Phosphatidylinositol-4-kinase type II β (PI4KIIβ) are not (Alfonso Pecchio et al., 2011; Cardozo Gizzi et al., 2015). As can be seen in the activity assays shown in Figure 3B, activation is achieved by increasing their V_max_ without affecting their K_m_. Furthermore, for activation to occur, c-Fos interacts with the enzymes it activates but not with those it does not regulate (see FRET experiments in Figure 3A). Interestingly, this physical interaction is achieved by the N-terminal domain of c-Fos, where a stretch enriched in basic amino acids (BD domain, 139–159) is of fundamental importance for enzyme activation (Alfonso Pecchio et al., 2011). Surprisingly, c-Fos can also perform this activity in the nucleus, where it promotes transcriptional changes in genes lacking an AP-1 consensus sequence in their promoters by increasing the nuclear content of phosphatidylinositol-4,5-bisphosphate (Ferrero et al., 2014). These findings provide an even more complex scenario regarding the cellular consequences of inducing c-Fos expression and allowed us to propose an additional role for c-Fos as a transcriptional regulator but in an AP-1-independent manner.

A great effort was destined to study this function in the nervous system, where c-Fos-dependent lipid activation has been consistently confirmed. In fact, the first studies that gave the initial evidence of this phenomena in the nervous system were performed in the chicken retina, where we found that during sensory stimulation, the transcription and transduction of c-Fos participate in the regulation of glycerophospholipids synthesis: if c-Fos expression is specifically inhibited in photoreceptors and ganglion cells, the differences usually observed in lipid synthesis after the exposure of the animals to light or dark cycles are abolished (Guido et al., 1996; Bussolino et al., 1998).

Later studies showed that this c-Fos-dependent lipid synthesis activation was also present in T98G cells, cells derived from a malignant human glioblastoma: blocking c-Fos expression in these cells promotes a decrease in the metabolic labeling of phospholipids and an impairment in cell proliferation (Portal et al., 2007; Ferrero et al., 2012). Results obtained in PC12 cells, which derive from a pheochromocytoma of the rat adrenal medulla, highlighted the existence of the dual functions of c-Fos as an AP-1 transcription factor and as an activator of lipid synthesis. Normally, the addition of nerve growth factor (NGF) to the cell culture media triggers a genomic differentiation program that drives these cells towards a sympathetic neuron phenotype, a process that is accompanied by an induction of c-Fos expression (Gil et al., 2004). If, together with NGF treatment, the entrance of c-Fos to the nucleus is impaired by blocking AP-1 importation, neuritogenesis and differentiation are inhibited even when c-Fos expression remains at its normal levels. However, if the import of c-Fos into the nucleus is blocked 16 hours after NGF induction, differentiation is normal. This implies that c-Fos is required at early stages of differentiation in the nucleus as AP-1 to trigger the differentiation program. However, once the cells have been induced to differentiate, when c-Fos expression is blocked, there is a decrease in lipid synthesis and neurites not only stop growing, they also begin to retract, a process that can be reverted by the over-expression of only c-Fos even in the absence of NGF. This indicates that c-Fos is no longer required in the nucleus at advanced times of differentiation but is rather required in the cytoplasm activating lipid synthesis at the ER to sustain neurite outgrowth (Gil et al., 2004).

**FIGURE 4:**
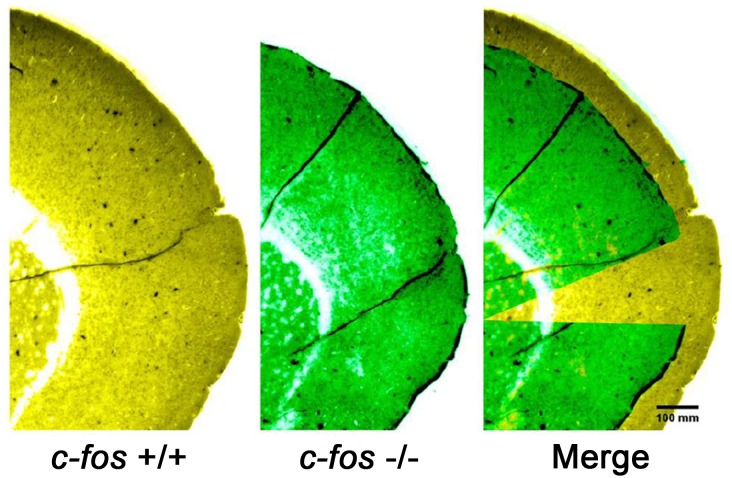
Representative images of brain cortical slices from *fos* (+/+) mice (left, pseudo colored yellow), *fos* (–/–) mice (middle, pseudo colored green) and the merged image (right). Note that in the merged image, part of the *fos* (–/–) image was cropped to better show the superposition of both images. Adapted from Velazquez et al., 2015b (this is an open-access article distributed under the terms of the Creative Commons Attribution License, which permits unrestricted use, distribution, and reproduction in any medium, provided the original author and source are credited).

To advance in the understanding of the role of c-Fos on brain development, studies were performed using transgenic *fos* (-/-) mice. These animals show a significant reduction in the thickness of the developing neocortex respect to wild type animals, a phenotype that is still observed at the adult stage (Figure 4; Velazquez et al., 2015a,b). This reduction correlates with a decrease in the number of differentiating cells and an increase in apoptosis levels at the ventricular zone. No differences are observed in the number of cells going through mitosis although the mitotic angle found is mainly vertical, suggesting a decreased tendency for the progenitor cells to differentiate. These results probably reflect the initial dependence of c-Fos AP-1 activity for neuronal development to occur.

**FIGURE 5:**
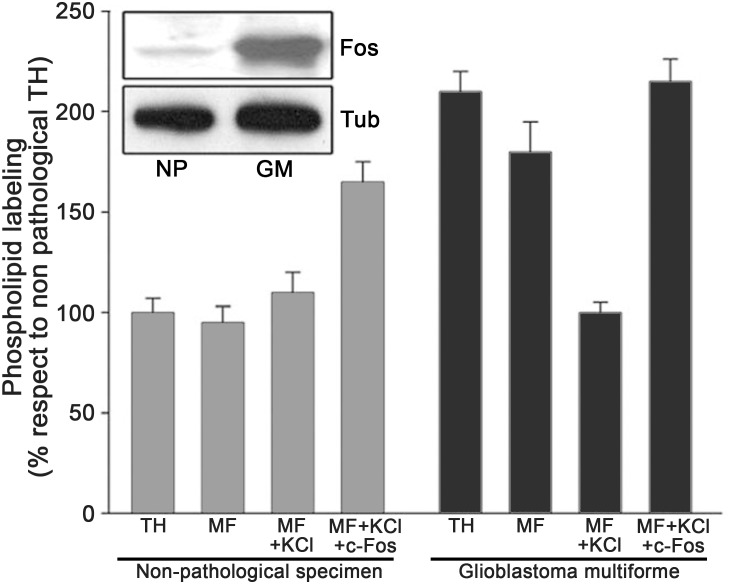
Activation of phospholipid synthesis in membranes from a human glioblastoma multiforme (GM). Homogenates were obtained from a human GM and from adjacent non pathological tissue (NP) excised from the same patient. TH: total homogenate; MF: microsomal membrane fraction; MF+KCl: 1M KCl stripped microsomal membrane fraction; MF+KCl+c-Fos: stripped MF plus recombinant c-Fos. Results are the mean ± SD of three experiments performed in triplicate; non-pathological TH values were taken as 100%. Inset: Western Blot for c-Fos content in the samples assayed; Tubulin staining is used as a loading control. Note the high content of c-Fos in the GM sample as compared to the almost undetectable levels in the NP sample. Adapted from Silvestre et al., 2010 (this is an open-access article distributed under the terms of the Creative Commons Attribution License, which permits unrestricted use, distribution, and reproduction in any medium, provided the original author and source are credited).

As c-Fos has been shown to be a potent oncoprotein that induces cell transformation when deregulated (Piechaczyk and Blanchard, 1994; Tulchinsky, 2000), we evaluated if this role could be due to its cytoplasmic lipid-synthetizing function. We found a strong expression of cytoplasmic c-Fos co-localizing with ER markers in 100% of the more than 200 human malignant brain tumor samples examined. This strongly contrasted with the lack of detectable expression of this protein in normal, paired biopsies (Silvestre et al., 2010). In a human glioblastoma multiforme sample, in addition to the increased expression of c-Fos, a > 100% activation of lipid synthesis was observed when assayed *in vitro*, with respect to a paired non-pathological sample. Furthermore, elimination from the assay media of endogenous c-Fos (using high ionic strength followed by centrifugation) reduced the activated levels of lipid synthesis, whereas addition of recombinant c-Fos to the c-Fos-devoid assay media re-established lipid synthesis levels (Figure 5).

Confirmation of the dependence of tumor growth on c-Fos expression was obtained in NPcis mice, a mouse model of the human disease Neurofibromatosis Type 1. These animals spontaneously develop central and peripheral nervous system tumors. In these mice, tumor progression is slowed down or even stopped by blocking c-Fos expression, in concordance with an abrogation of phospholipid synthesis activation in spite that AP-1 content is not substantially affected (Silvestre et al., 2010). Strikingly, NPcis *fos* (−/−) mice do not develop tumors in contrast to their *fos* (+/−) or *fos* (+/+) littermates, in which tumor development was observed in 71% of the animals (Silvestre et al., 2010). These *in vivo* examples of the dependence on c-Fos for nervous system tumors to grow disclose c-Fos as a potential target to control brain cancer.

## Final Comments

All the above-mentioned results highlight the relevance of c-Fos for the development of the nervous system. The emergence of an AP-1-independent function that relies on the capacity of c-Fos to activate lipid synthesis provides an explanation for many results observed upon the induction of c-Fos expression that remained obscure even though almost 35 years have passed since the first description of this protein. To the best of our knowledge, c-Fos is the first transcription factor with the capacity to regulate lipid synthesis independently of its canonical transcriptional function. The SREBPs transcription factors, for example, are tightly linked to fatty acid and cholesterol synthesis but only through their genomic activity in the nucleus (Eberle et al., 2004; Cheng et al., 2018).

Many questions regarding the cytoplasmic function of c-Fos remain to be elucidated. Clearly, c-Fos expression is related to changes in cell cycle and morphology, controlling different aspects of development (Hess et al., 2004). The fact that c-Fos can act in two different ways, either as an AP-1 transcription factor or activating lipid synthesis, raises the interrogation about how these two functions are coordinated or which are the signals that trigger one or the other function to exert a physiological/pathological role. It has been proposed that AP-1 fulfills a homeostatic role in cells in response to changes in their environment and growth conditions, adjusting gene expression so as to allow the cell to adapt to those changes (Shaulian and Karin, 2001). If we now consider the function of c-Fos as a lipid synthesis activator, the perspective must necessarily be deepened. It seems reasonable to think that the elicited function will depend on the triggering stimulus. In any case, studies must be extended in order to further elucidate this novel mechanism.

c-Fos was the first transcription factor whose induction was shown to be dependent on neuronal activity (Morgan and Curran, 1988; Sagar et al., 1988). As stated previously, this attribute rapidly transformed c-Fos into a marker for neuronal activity. The increase in its expression was observed in the central nervous system after applying learning and memory recovery protocols (Gallo et al., 2018). Interestingly, these changes in c-Fos expression were observed mainly during the first training sessions, indicating that this is probably an adaptive response. Consequently, it seems reasonable to propose that, in light of the above mentioned evidence, this adaptive response might be entailed with neuronal and synaptic plasticity processes that involve the cytoplasmic function of c-Fos: once all the new circuits related to a specific training are formed and established, the cells require a lower amount of lipids just to maintain all the pre-formed contacts. In fact, a reduction in synaptic plasticity mechanisms was found in the hippocampus of a selective *fos* knock-out mouse (Fleischmann et al., 2003).

In any case, all the background information described in the literature regarding the role of c-Fos in the physiology of the nervous system consistently confirmed its importance for cellular plasticity although they have always been associated to changes in gene expression. But, could they be related to changes in lipid homeostasis? This hypothesis seems reasonable given the evidence underlined herein. In this regard, we are currently finishing additional studies on the role of c-Fos in neuronal differentiation, using primary cultures as development models. This will allow us to unravel the mechanisms through which c-Fos participates in normal brain development.

The apparently unrelated functions of c-Fos as a transcription factor or as an activator of lipid synthesis may be related to its structural plasticity given its nature as an Intrinsically Disordered Protein (IDP), a characteristic already described for other proteins (Tompa, 2005). IDPs do not have a tridimensional defined structure in their native states (Dyson and Wright, 2005). This feature gives them the advantage of having conformational plasticity and allows them to recognize and associate with multiple, different targets (Wright and Dyson, 2009). Even though we have described that the N-terminal of c-Fos together with its BD are responsible for its association with different enzymes and their subsequent activation, it is yet to be determined if c-Fos suffers a conformational change to an ordered structure during this process.

It is promising to consider the reaches of this new role of c-Fos in physiological and pathological aspects of the nervous system. It could be involved in controlling neuronal development and in the establishment of correct neuronal circuits, or in regeneration events that require high amounts of lipid synthesis for membrane biogenesis. More importantly, the possible participation of c-Fos in lipid homeostasis in the neurobiology of disease should be approached. Highlighting the importance of lipid synthesis activation by c-Fos is the observation that, by specifically blocking its expression, proliferation and growth of tumor cells of the nervous system are slowed or halted. This discloses the cytoplasmic activity of c-Fos as a potential target for controlling growth of brain carcinomas. Future studies are being driven through this exciting road.

## Author Contributions

Both authors participated in the preparation of the manuscript.

## Conflict of Interest Statement

The authors declare that the research was conducted in the absence of any commercial or financial relationships that could be construed as a potential conflict of interest.
